# Identification of clinical target areas in the brainstem of prion‐infected mice

**DOI:** 10.1111/nan.12189

**Published:** 2015-04-23

**Authors:** Ilaria Mirabile, Parmjit S. Jat, Sebastian Brandner, John Collinge

**Affiliations:** ^1^ MRC Prion UnitDepartment of Neurodegenerative DiseaseUCL Institute of NeurologyLondonUK

**Keywords:** brainstem, clinical target areas, cre‐*lox* system, locus coeruleus, neurodegeneration, prions

## Abstract

**Aims:**

While prion infection ultimately involves the entire brain, it has long been thought that the abrupt clinical onset and rapid neurological decline in laboratory rodents relates to involvement of specific critical neuroanatomical target areas. The severity and type of clinical signs, together with the rapid progression, suggest the brainstem as a candidate location for such critical areas. In this study we aimed to correlate prion pathology with clinical phenotype in order to identify clinical target areas.

**Method:**

We conducted a comprehensive survey of brainstem pathology in mice infected with two distinct prion strains, which produce different patterns of pathology, in mice overexpressing prion protein (with accelerated clinical onset) and in mice in which neuronal expression was reduced by gene targeting (which greatly delays clinical onset).

**Results:**

We identified specific brainstem areas that are affected by prion pathology during the progression of the disease. In the early phase of disease the locus coeruleus, the nucleus of the solitary tract, and the pre‐Bötzinger complex were affected by prion protein deposition. This was followed by involvement of the motor and autonomic centres of the brainstem.

**Conclusions:**

Neurodegeneration in the locus coeruleus, the nucleus of the solitary tract and the pre‐Bötzinger complex predominated and corresponded to the manifestation of the clinical phenotype. Because of their fundamental role in controlling autonomic function and the overlap with clinical signs in sporadic Creutzfeldt–Jakob disease, we suggest that these nuclei represent key clinical target areas in prion diseases.

## Introduction

The most frequent form of human prion disease, sporadic Creutzfeldt–Jakob disease (sCJD) typically presents as a rapidly progressive multifocal dementia. Typically, patients progress to akinetic mutism and death within 4 months. Frequent neurological features include myoclonus, extrapyramidal signs, cerebellar ataxia, pyramidal signs and cortical blindness. Some of the clinical signs suggest brainstem involvement, for example dysphagia, dysarthria, respiratory failure and ophthalmoplegia. Histologically, affected areas show evidence of neurodegeneration with vacuolation (spongiosis) [1, 2, 3], the pathogenesis of which is still debated. It may arise from abnormal membrane permeability and increased water content in the neurones [4], from autophagy [5], or may be due to production of abnormal prion protein (PrP) within the lysosomal compartment, which would cause disruption of the lysosomal membrane, destruction of the neuronal cytoskeleton and initiation of vacuolation [6]. Other histological features are loss of neurones, invariably accompanied by astrocytic proliferation (gliosis) and microglial activation. Usually these findings correlate with the deposition of abnormal (disease‐associated) prion protein. According to the protein‐only hypothesis [7, 8], prions consist of multichain forms of misfolded host‐encoded cellular prion protein (PrP^C^), referred to as PrP^Sc^, and propagate by an autocatalytic process of seeded fibrillization [9, 10]. It is still unclear how prion propagation results in neurodegeneration and clinical onset. Prion propagation and neurotoxicity can be uncoupled in animal models; prions themselves may not be directly neurotoxic [11, 12], and neurotoxicity may relate to production of toxic species distinct from the propagating infectious species but catalysed by them [9]. Infection with a defined prion strain in a distinct inbred mouse line is associated with prolonged but remarkably consistent incubation periods followed by a very rapid clinical phase. Recent work measuring the kinetics of prion propagation in mice with different levels of PrP^C^ expression described two distinct mechanistic phases during the clinically silent incubation period.

In the first phase, prions propagate exponentially, not rate‐limited by PrP^C^ concentration, until a defined maximal titre is reached, arguing that infectious prions comprise a small minority of available PrP. In the second plateau phase, there is a linear rise in Proteinase K (PK) sensitive disease‐related PrP isoforms, a mechanistically distinct process rate limited by, and directly proportional to, PrP^C^ concentration. Clinical onset occurs at a similar level of PK‐sensitive disease‐related PrP isoforms, irrespective of PrP^C^ expression level. Prion titres are essentially constant, and the length of this plateau to clinical onset is inversely proportional to PrP^C^ expression level [13, 14]. It is argued that a toxic species PrP lethal (PrP^L^) [12, 15] is formed in this second phase by a process linearly related to PrP^C^ concentration, and neurodegenerative changes occur when PrP^L^ reaches a local toxic threshold. The histopathological correlate of the preclinical phase is a mild accumulation of abnormal PrP, activation and proliferation of astrocytes [16, 17, 18] and microglia cells [19, 20]. The progression to the clinical phase is characterized by additional synaptic loss and increasing spongiform degeneration, followed by neuronal death [21].

However, it is still not clear how the distribution of prion disease‐related pathology in different brain regions relates to the onset and progression of disease. Earlier studies focused on the effect of different routes of infection on the clinical phase of prion disease. It was reported that the scrapie replication phase in the brain was shorter after peripheral administration of prions (intraperitoneal, or i.p.), than after direct intracerebral inoculation (i.c.) in the forebrain [22, 23, 24]. This observation led to the postulation that the development of clinical prion disease depends on prion infection spreading to specific ‘clinical target areas’ resulting in cell dysfunction and cell death. It was therefore speculated that the duration of the ‘neural’ phase of pathogenesis is determined by the complexity of the pathways between the entry site of infection (injection site) and the postulated clinical target areas in which scrapie should replicate for the disease to develop [22]. Clinical signs highly suggestive of brainstem involvement are seen in experimental rodent models of prion disease: brainstem nuclei control essential body functions such as motor control, generation of the respiratory rhythm and regulation of the blood pressure, and indeed, unsteady gait is an early clinical manifestation and abnormal respiration is a key late feature.

In humans, widespread PrP deposition in the brainstem has been reported as an early pathologic event in sCJD [25]. This study correlated clinical signs and histopathological changes in 33 sCJD brainstems. It showed high variability among samples and was unable to determine a correlation between clinical signs and brainstem lesions. However, brainstem atrophy, neuronal loss, pyramidal tract degeneration and PrP deposition were reported, particularly in patients with prolonged disease. The authors speculated that a conclusive evaluation on the relation between clinical signs and brainstem impairment is difficult because the same symptoms could derive from overlapping involvement of basal ganglia or cortex [25].

Mouse models have the advantage of avoiding the variability of human pathology and to directly correlate the temporal relationship between onset of clinical signs and brainstem involvement. In previous studies, mice in which neuronal PrP^C^ expression was ablated during established CNS infection with the Rocky Mountain Laboratories (RML) prion strain were significantly protected against onset of clinical disease [26]. We repeated these studies using both RML and ME7 prion strains in single transgenic mice with overexpression of PrP^C^ (M*lox*P line) and double transgenic mice in which the PrP transgene can be deleted by Cre‐mediated excision (NFH‐Cre/M*lox*P line), to see if we could relate onset of brainstem pathology to clinical onset in these models with different pathogenesis and incubation periods. Previous studies have shown that the first manifestation of prion pathology occurs in the thalamus and brainstem in PrP^C^ overexpressing RML prion‐inoculated [27] and ME7 prion‐inoculated mice (Figure S3). Double transgenic mice are particularly useful for this study as they have a mild and significantly protracted disease progression, which will allows us to study the localized accumulation of abnormal PrP and the developing pathological changes at a higher temporal resolution [26, 28, 29]. Also, we aimed at a comparison with previously characterized models and their kinetics [26].

Here, we set out to systematically investigate the putative clinical target areas of prion disease by a comprehensive analysis of the evolution of brainstem pathology in these models [27]. We observed early colonization of specific nuclei in the brainstem and followed the disease progression in these areas. Although the thalamus showed early signs of prion protein deposition, we specifically investigated the brainstem, where the nuclei play a role in controlling autonomic functions and survival. We propose that loss of neuronal tissue within the brainstem nuclei may explain some of the clinical symptoms in terminal prion disease and suggest that they may represent specific clinical target areas for prion disease. The critical role of these nuclei in maintenance of essential neurological function may explain the rapidity of clinical decline in some animal models and human prion diseases.

## Materials and methods

### Experimental animals

All animal experiments conformed to United Kingdom regulations and institutional guidelines and were performed under Home Office project license (PPL70/6454).

M*lox*P and NFH‐Cre/M*lox*P transgenic mice were generated as described in detail previously [26, 29] on a *Prnp^0/0^* background where PrP is expressed under the control of a hemizygous PrP (M*lox*P) transgene, on an inbred FVB/N background. These mice show a decreasing expression of PrP^C^ at 8–10 weeks due to a late onset NFH‐cre mediated removal of PrP expression. ROSA26 reporter mice for cre activity (ROSA26*^Lox^*) were used to detect Cre‐mediated recombination [30]. ROSA26*^Lox^* homozygous mice were crossed to homozygous NFH‐Cre mice, to obtain hemizygous mice for both transgenes (LacZ and NFH‐Cre) to be used for β‐galactosidase immunohistochemistry.

NFH‐Cre/M*lox*P mice result from the cross of M*lox*P mice and NFH‐Cre mice, expressing Cre recombinase under control of Neurofilament Heavy Chain promoter [31], which targets expression to adult neurones [29]. In double transgenic mice, the Cre enzyme excises the PrP coding sequences placed within the two *lox*P sites, effectively eliminating PrP expression in the recombined cells [29]. Thus, double transgenic NFH‐Cre/M*lox*P mice express PrP in neurones and non‐neuronal cells until 8–12 weeks of age, when they undergo Cre‐mediated depletion of neuronal PrP^C^. Also the efficacy of recombination varies between brain regions, explaining the sustained production of PrP^SC^ while survival is significantly prolonged [26]. These transgenic animals were chosen over wild type strains as they allow for a detailed, anatomically selective functional and anatomical‐pathological analysis and correlation. Mice were individually tagged with subcutaneous transponders for unequivocal identification and housed in a temperature‐ and light‐controlled mouse room (12 h light/dark cycles) in groups of four to six mice. All mice had free access to food and water.

Mice were examined daily for early signs of prion disease and culled when defined diagnostic clinical signs occurred, or time‐culled at 6 and 12 (RML infection) or 8 and 16 (ME7 infection) weeks post‐inoculation (wpi). All brains were collected and processed for histology, of which three or more mice per group were analysed in detail histologically. Cohorts of at least 10 mice of the genotypes M*lox*P and NFH‐Cre/M*lox*P were inoculated with RML or ME7 and were observed until scrapie end stage to determine survival curves or were time culled. Mice dying within 4 weeks of the inoculation were excluded from the analysis.

### Neurological observation

Following inoculation with RML or ME7 prions, mice were examined daily for appearance of scrapie symptoms or other signs of illness. Early indicators of prion disease include erect ears, rigid tail, piloerection and ungroomed appearance; slightly hunched posture; and clasping of hind limbs when lifted; confirmatory signs include ataxia, generalized tremor, impaired breathing activity, loss of righting reflex or limb paralysis. The clinical signs that defined an experimental mouse as terminally ill are suggestive of brainstem malfunction affecting both motor control (unsteady gate, tremor and ataxia) and respiratory control (abnormal breathing rate). For end‐point experiments, animals were culled as soon as clinical prion disease was confirmed or if they showed signs of distress or loss of 20% of body weight. All symptoms were recorded on videotape prior to culling of the animal.

### Preparation of prion inocula and inoculation of animals

Hemizygous M*lox*P and NFH‐Cre/M*lox*P mice were inoculated with Rocky Mountain Laboratories (RML) or ME7 prion strains. RML (MRC Prion Unit Inoculum reference I8700) and ME7 (I9458) prion inocula were prepared as a 10% (w/v) brain homogenate in Dulbecco's phosphate buffered saline lacking Ca^2+^ or Mg^2+^ ions (D‐PBS) and titred by bioassay in Tg20 mice (19). Prion titres were calculated using the Reed–Muench formula (I8700 titre = 8.2 Log LD50/g brain; I9458 titre = 9 Log LD50/g brain). I8700 and I9458 were diluted to 1% (w/v) with D‐PBS and 20 μl of 1% brain homogenate were inoculated intracerebrally into the right parietal lobe of anesthetized 1‐week‐old mice. The inoculation at this early time point was necessary to allow for development of prion disease before the onset of recombination and loss of PrP expression in the CNS as described previously [26, 28, 29]. Following intracerebral inoculation, prions are distributed widely in the CNS rather than in a specific location [24, 27, 32], but eventually pathological changes appear in specific areas. In the mouse strains used here, the first two areas of prion replication and detection of abnormal prion protein, independent of the precise inoculation site, are the thalamus and the dorsal brainstem.

### Histology

Brains from time‐culled or terminally ill mice were fixed in 10 % buffered formal saline. Brains were dissected on the level of the striatum, the anterior hippocampus/thalamus and the midbrain with the rostral end of the cerebellum. Each of the slices was processed separately and embedded in paraffin wax. Serial coronal sections from the most caudal block, corresponding to the brainstem and the cerebellum, were cut at a nominal thickness of 3 μm, with five consecutive serial sections, followed by a 50 μm gap between levels, to allow a reproducible and consistent analysis of all brainstem regions. Brain sections were stained with haematoxylin‐eosin and prepared for immunohistochemical staining. Antibodies or antisera against the following antigens were used: PrP (ICSM35, D‐Gen London UK), glial fibrillary acidic protein (GFAP) (DAKO Z0334 Ely, UK), β‐galactosidase (Abcam Ab616 Cambridge, UK), ionized calcium‐binding adapter molecule 1 (Iba‐1) (Abcam 5076) and Neurokinin 1 (NK1) receptor (Pierce PA1‐32229 Hemel Hempstead, UK). All immunostainings were carried out on the automated Ventana Benchmark XT or Discovery XT (Roche Burgess Hill, UK) staining instrument, using biotinylated secondary antibodies and a horseradish peroxidase‐conjugated streptavidin complex and diaminobenzidine as a chromogen.

### Semi‐quantitative neuropathological analysis and region mapping

Sections of brains from time‐culled mice and from those succumbing to scrapie were examined blind by the same person. Sections from prion‐infected mice were semi‐quantitatively assessed for spongiosis, abnormal PrP accumulation and gliosis, on a scale from 0 to 3, but intermediate scores were given when appropriate (Figure S1). Spongiform changes were scored as follows: ‘mild’ corresponds to occasional vacuoles, not exceeding two large vacuoles in a high power field (HPF; 40× objective), and generally absent or minimal microvacuolation in the same field (Figure S1A), ‘moderate’ or ‘intermediate’ indicates up to five large vacuoles, often accompanied by a mild microvacuolation in the same field (Figure S1B) and ‘severe’ indicates more than five vacuoles, often accompanied by a more widespread microvacuolation in the same field (Figure S1C). Scoring of the deposition of prion protein, included in addition to the overall density and intensity, the quality of the stain that is synaptic (Figure S1D–F) or granular (Figure S1G–I). ‘Synaptic’ deposition was defined by a fine, diffuse and homogenous immunolabelling with no formation of granules, irregularities or aggregates. A score of ‘mild’ was given when the immunolabelling showed a fine, homogenous labelling as shown in Figure S1D. A stronger labelling with a slight accentuation of neuronal bodies was graded as ‘intermediate’ or moderate (Figure S1E) and a robust labelling reaching saturation of immunostaining was graded as ‘strong’ (Figure S1F). ME7 inoculum was associated with the formation of coarse granules, and therefore a separate scoring was applied. A ‘mild’ score was given when occasional granules were seen against a mild synaptic background, as shown in Figure S1G. A more widespread presence of granules, which often increased in size, associated with a stronger synaptic background were scored as ‘intermediate’, as shown in Figure S1H. Strong immunolabelling, with frequent coarse grains, decorating the majority of neurones in the area, sometimes nearly confluent were scored as ‘strong’ (Figure S1I). Gliosis, assessed by immunolabelling of GFAP was scored ‘mild’ if reactive astrocytes, separated by non‐reactive neural parenchyma, were identified as shown in Figure S1J. A more dense labelling, with immunoreactivity covering most or all of the intervening neural parenchyma, was scored as intermediate (Figure S1K), and the presence of severely immunoreactive, hypertrophic astrocytes was scored as ‘severe’ gliosis (Figure S1L). The intensity and density of microglia staining, carried out on a subset of sections, matched that of GFAP immunolabelled astrocytes. Because it did not provide additional information, the scoring and correlation with other features was therefore not carried out.

**Figure 1:**
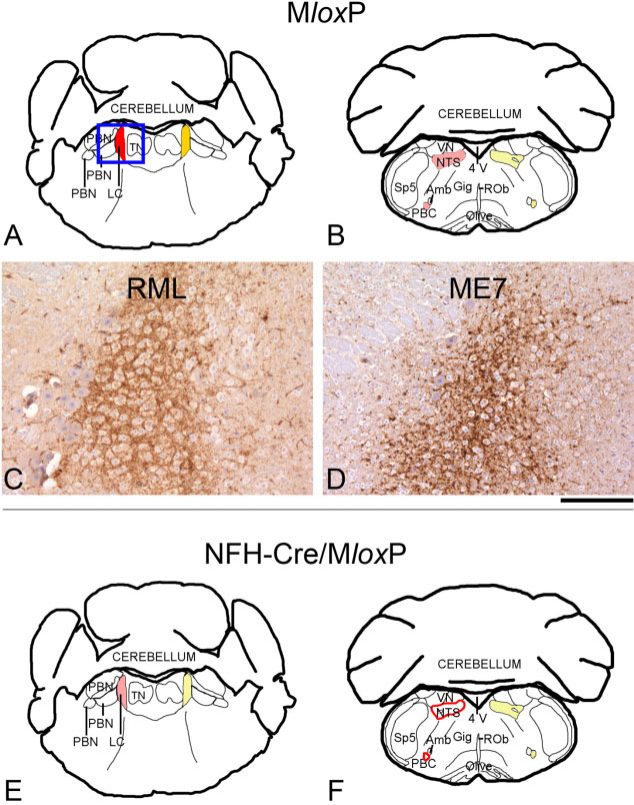
Abnormal PrP accumulation in the locus coeruleus of RML‐ and ME7‐inoculated M*lox*P and NFH‐Cre/M*lox*P mice. Early prion pathology in the brainstem of RML and ME7 prion‐inoculated M*lox*P (**A–D**) and NFH‐Cre/M*lox*P (**E**,**F**) mice, time‐culled at an early asymptomatic stage (RML, 6 wpi; ME7, 8 wpi), manifested with deposition of disease‐associated prion protein (red = intermediate, pink = mild, red outline = very mild) and reactive gliosis [dark yellow = intermediate (**A**); light yellow = mild (**B**,**E**,**F)**] in the locus coeruleus (LC), the nucleus of the solitary tract (NTS) and the pre‐Bötzinger complex (PBC). No spongiform changes were observed at this time point. (**A**,**B**,**E**,**F**) Schematic representations adapted from the Paxinos Mouse Atlas, −5.34 mm (**A**,**E**) and −6.84 (**B**,**F**) from Bregma. (**C**,**D**) Synaptic PrP deposition in the LC, in RML‐inoculated M*lox*P mice (**A**), and granular deposits in ME7‐inoculated M*lox*P mice (**D**). 4 V, fourth ventricle; Amb, nucleus ambiguous; LC, locus coeruleus; NTS, nucleus of the solitary tract; PBC, pre‐Bötzinger complex; PBN, parabrachial nuclei; ROb, raphe obscurus; Sp5, spinal nucleus of the V nerve; TN, tegmental nuclei; VN, vestibular nuclei. Scale bar below D = 160 μm.

Identification of brainstem nuclei was carried out by using corresponding tables of the Paxinos and Franklyn mouse atlas based on stereotaxic coordinates. The following areas were consistently identified and scored for spongiosis, PrP load and quality and gliosis: vestibular nuclei (VN), prepositus nucleus (PRN), olive, cerebellum, latero‐dorsal tegmental nuclei, locus coeruleus (LC), parabrachial nuclei (PBN), gigantocellular reticular nucleus (GIG), nuclei of the solitary tract (NTS), ventrolateral reticular medulla (VLRM), pre‐Bötzinger nucleus (PBC) and the nucleus of the sensory portion of the trigeminal nerve (N CN5). Scoring results of at least three animals were collated on a spreadsheet (MS Excel 2010; Figure 4). Scores were averaged and cross‐referenced on a separate worksheet in the same file and visually enhanced by colour codes using the conditional formatting function. To calculate the intensity variance between spongiosis, gliosis and prion protein deposition, standard deviation (SD) was calculated. A small value indicates a parallel development of the three pathologies while a high value indicates a discrepancy between the different features. Again, for visual enhancement this was colour coded using the conditional formatting function. Similarly, variability of each of the pathologies (e.g. prion protein intensity) across different regions was estimated by calculating the SD across all regions (Figure 1, Figure 4 Column **Q**). Photographs were taken with a ColorView II digital camera (http://www.soft‐imaging.de Olympus Soft Imaging Solutions GmbH Münster, Germany) mounted on a ZEISS Axioplan microscope (Carl ZEISS ltd, Cambridge, UK) and composed with Adobe Photoshop (Adobe Systems Europe Ltd, Maidenhead UK).

## Results

### Clinical signs suggestive of brainstem involvement, incubation times and survival of mice

**Figure 2:**
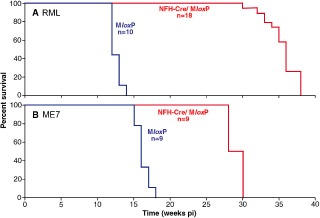
Survival of RML‐ and ME7‐ inoculated M*lox*P and NFH‐Cre/M*lox*P mice. Mice were inoculated with RML or ME7 prions at 1 week of age. M*lox*P mice (blue line) became terminally ill at approximately 12 wpi (RML, **A**) or at approximately 17 wpi (ME7, **B**). In contrast to previous experiments, recombined NFH‐Cre/M*lox*P mice (red) ultimately developed prion disease at approximately 35 weeks (**A**) or approximately 29 weeks (**B**) but both showed significant delay of their incubation time (*P* < 0.001).

Brainstem nuclei control essential body functions, such as motor control, generation of respiratory rhythm and regulation of blood pressure. In keeping, typical signs that were observed and monitored in mice with preterminal and terminal illness were unsteady gate, tremor, ataxia and abnormal breathing rate. In early and mid‐progression phases, stimulus sensitive cloni and/or startle reactions could also be evoked. Towards the final disease stage, animals presented with signs of a spastic paraplegia, often accompanied by sustained irregular motor hyperactivity, interrupted by unresponsive and akinetic intervals and the development of a slight disturbance that progressed to a complete loss of the animal's natural resting‐activity cycle, indicative of impairment of the reticular formation. RML‐inoculated M*lox*P mice (*n* = 10) survived 12.6 weeks (range 12–14 weeks, median 12 weeks), ME7‐inoculated M*lox*P mice (*n* = 9) survived 16.2 weeks (range 15–17, median 16 weeks), whereas the ‘recombined’ counterparts, NFH‐Cre/M*lox*P mice, survived for 34.7 weeks after RML inoculation (median 35, range 30–38 weeks) and 28.9 weeks after ME7 inoculation (median 28, range 29–30 weeks). The survival difference between RML and ME7 inoculum was statistically highly significant for both genotypes (RML in NFH‐Cre/M*lox*P *vs.* M*lox*P and ME7 NFH‐Cre/M*lox*P *vs.* M*lox*P *P* < 0.001), indicating that Cre‐mediated PrP recombination significantly prolonged the incubation time of prion‐infected mice (Figure 2).

### Areas of pathology in asymptomatic prion‐infected mice (6–8 wpi)

Detailed analysis of pathological changes in the brainstem of PrP overexpressing (M*lox*P) and PrP‐depleted (NFH‐Cre/M*lox*P) mice at early asymptomatic stages (RML, 6 wpi; ME7, 8 wpi) was done on coordinates ranging from −5.34 mm to −7.64 mm, and included brainstem nuclei involved in gait and balance (VN, PRN and olive); and autonomic activity (LC, latero‐dorsal TN, PBN, GIG, NTS, VLRM and PBC). These areas were chosen because motor activity and autonomic functions (that is respiration) are usually impaired in terminally ill mice. The cerebellum is connected with the brainstem through the olivo‐cerebellar pathway, and was analysed to confirm and characterize in greater detail a previous, unpublished observation of late cerebellar pathology in longer surviving terminally ill ME7‐infected NFH‐Cre/M*lox*P mice.

**Figure 3:**
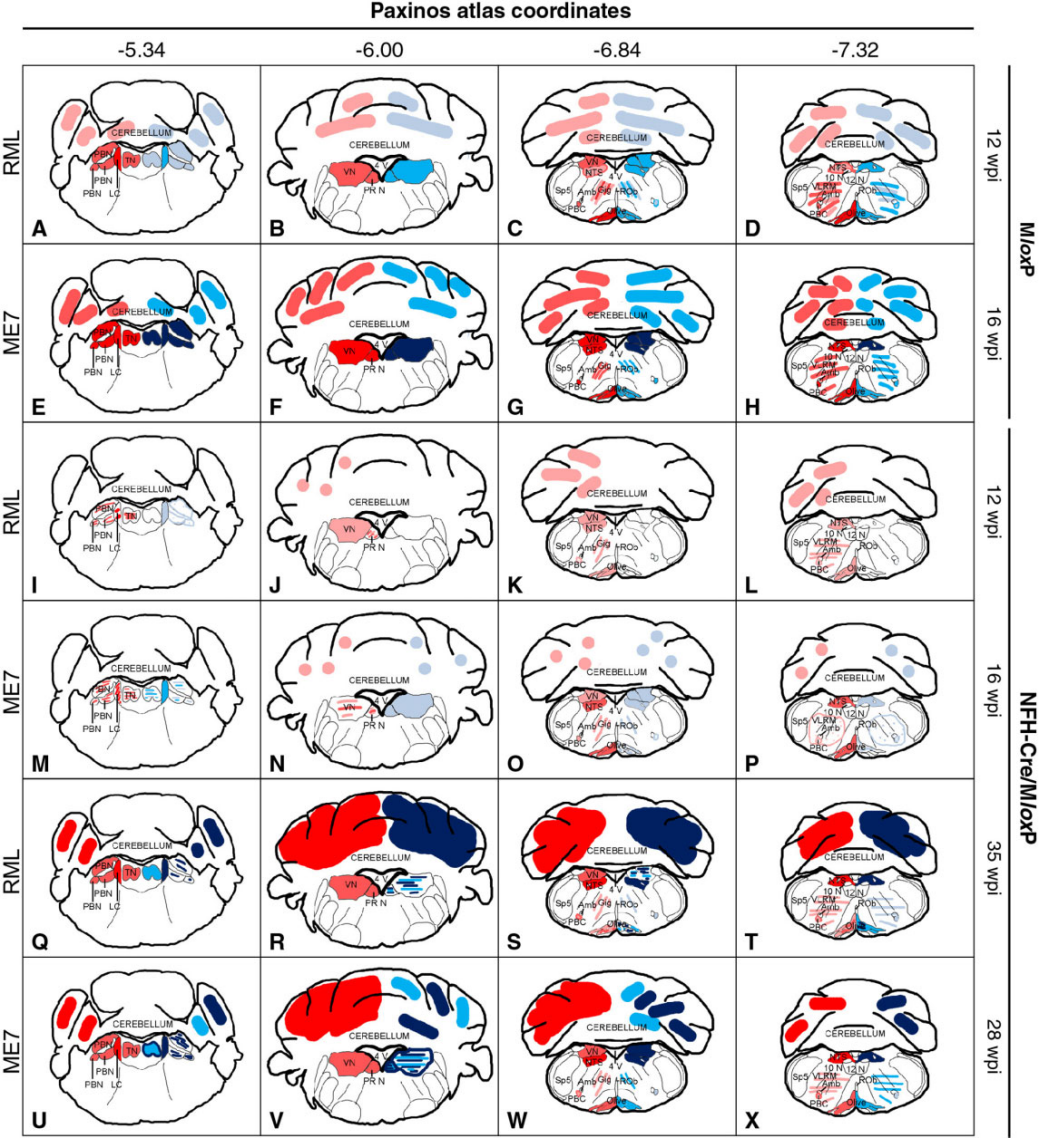
Progression of prion pathology in RML‐ and ME7‐inoculated M*lox*P and NFH‐Cre/M*lox*P mice. Schematic drawings of abnormal PrP deposition (pink = mild, red = intermediate, dark red = severe) and spongiosis (pale blue = mild, blue = intermediate, dark blue = severe) of the brainstem nuclei analysed, at four representative levels from coordinates −5.34 mm to −7.64 mm caudal from bregma. The inoculum is shown on the left margin. Genotype and incubation times on the right margin. (**A–D**) In terminally ill RML‐inoculated M*lox*P mice (12 wpi) PrP deposition is seen in different areas of the brainstem, including the locus coeruleus and the olive; prepositus nuclei and tegmental nuclei; and pre‐Bötzinger complex; spongiosis corresponds to PrP deposition in most areas. (**E–H**) In terminally ill ME7 inoculated M*lox*P mice (16 wpi), abnormal PrP deposition was severe in all the nuclei examined, except the gigantocellular nucleus, the ventral medulla and the cerebellum, where it was intermediate. Spongiosis pattern corresponds to PrP deposition in most areas. (**I–L**) RML‐inoculated NFH‐Cre/M*lox*P mice show a significantly lower overall accumulation at 12 wpi, and spongiosis was nearly absent everywhere, except for the locus coeruleus, where it was mild (pale blue). (**M–P**) In ME7‐inoculated NFH‐Cre/M*lox*P mice culled at 16 wpi, abnormal PrP accumulation was slightly stronger than in RML‐inoculated animals and more heterogeneous. Spongiosis was reduced everywhere compared with ME7‐inoculated M*lox*P mice at end stage of prion disease. Strong PrP deposition and spongiosis was observed in terminally ill RML‐inoculated NFH‐Cre/M*lox*P mice (35 wpi, **Q–T**), or in terminally ill ME7‐inoculated NFH‐Cre/M*lox*P mice (28 wpi) (**U** **–X**). 10 N, nucleus of the X nerve; 12 N, nucleus of the XII nerve; 4 V, forth ventricle; Amb, nucleus ambiguous; LC, locus coeruleus; NTS, nucleus of the solitary tract; PBC, pre‐Bötzinger complex; PBN, parabrachial nuclei; PR N, prepositus nucleus; ROb, raphe obscurus; Sp5, Spinal nucleus of the V nerve; TN, tegmental nuclei; VLRM, ventro‐lateral reticular medulla; VN, vestibular nuclei; VN, vestibular nuclei.

**Figure 4:**
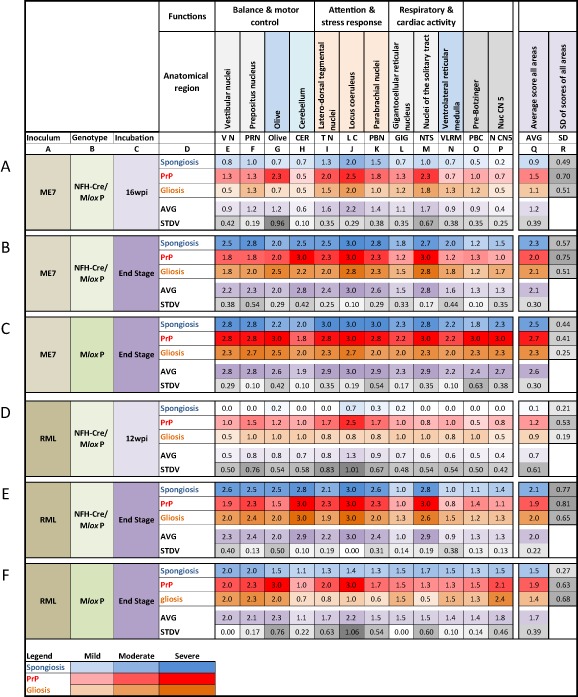
Summary scores from multiple individual experiments. The top two rows (unlabelled) show the anatomical brainstem regions and their function. Rows **A–F** show the experimental groups, by inoculum, genotype and incubation time. Above row **A**, columns **A–R** provide reference points for the in text citations of anatomical regions. Each of the rows **A–F** display the summary scores for spongiosis (blue), PrP deposition (red) and gliosis (orange), averaged from at least three experimental animals. The colour intensity is directly proportional to the average scores and was computed with the ‘conditional formatting’ in MS Excel. Below the individual pathology scores, an average of these values is formed to inform about the overall (average or aggregated) pathology. In addition, the row below this average, the standard deviation is calculated to indicate low or high variation between the three scores. A low score (light grey) indicates that all three pathologies develop synchronously in a given region, whereas a high score (dark grey) indicate that the three pathologies develop asynchronously. On the right, columns Q and R indicate the average and standard deviation across all regions. A low value indicates that all regions show similar pathology across the entire brainstem (homogenous) and a high value indicates that some areas show a stronger deposition than others. The legend on the bottom indicates the colours corresponding to scores mild (value 1), moderate (2) and severe (3).

In M*lox*P mice at 6 wpi (RML) or 8 wpi (ME7), PrP accumulation (Figure 3 **C**,**D**) and gliosis, but no spongiosis were seen in the LC, the NTS and the PBC, identified by NK1 receptor immunoreactivity. At this stage a subtle prion deposition pattern difference between ME7‐inoculated (coarse granular) and RML‐inoculated mice (fine synaptic) was seen (Figure 3 **C**,**D**). Also in NFH‐Cre/M*lox*P mice, the LC, NTS and PBC were the only nuclei showing prion pathology but at a reduced intensity compared with the non‐recombined M*lox*P mice (Figure 3 **E**,**F**). Cre‐mediated recombination prolongs survival of prion‐infected mice and slows disease progression in the LC, the nucleus of the solitary tract and the PBC. To understand the progression of brainstem pathology, we analysed RML‐ and ME7‐inoculated M*lox*P mice at late stages of prion disease (RML, 12 wpi; ME7, 16 wpi); and NFH‐Cre/M*lox*P mice at intermediate (RML, 12 wpi; ME7, 16 wpi) or late (RML, 35 wpi; ME7, 28 wpi) stages of prion disease (Figure 4).

### Prion pathology in terminally ill, non‐recombined M*lox*P mice

Clinically ill M*lox*P mice (RML, 12 wpi; ME7, 16 wpi) showed an extensive deposition of abnormal PrP in the brainstem. RML‐inoculated mice showed more variation across the different areas (SD 0.63) than those inoculated with ME7 [(SD = 0.41), Figure 1 **C**,**F**]. RML inoculation caused strong deposition in the LC and the olive; followed by intermediate intensity in the parabrachial, vestibular, prepositus, tegmental, nuclei, NTS and PBC; and mild accumulation in the CER and the VLRM (Figure 4 **A–D** and 4 **F**). Instead, ME7‐inoculated mice showed a more uniform deposition in these areas of the brainstem (Figures 4 **E–H** and 4 **C**). Both groups showed prominent spongiform changes and gliosis, which was proportional to the degree of PrP deposition. However, RML inoculation resulted in a finer vacuolation pattern (scored as ‘intermediate’), whereas in ME7‐inoculated brains the vacuoles were considerably larger (scored as ‘severe’) (Figures 4 **A–D**,**E–H** and S1).

### Prion pathology in recombined NFH‐Cre/M*lox*P mice: Differences between RML and ME7 inocula

At the time points when the M*lox*P mice succumb to disease (RML, 12 wpi; ME7, 17 wpi) recombined NFH‐Cre/M*lox*P mice showed very mild prion pathology, which extended to most grey matter areas in the brainstem (Figure 4 **I–L**,**M–P**, Figure 1, Rows **A**,**D**). In both RML‐ and ME7‐inoculated NFH‐Cre/M*lox*P mice, the LC showed the most intense abnormal PrP deposition (Figure 1, Column **J**), followed by the olive, PBN and TN (Figure 1, Columns **G**,**I**,**K**). RML produced minimal spongiform degeneration (average score 0.1), and gliosis (average score 0.9) (Figure 1, Row **D**, Column **Q**) while ME7‐infected mice showed mild spongiosis and gliosis in almost all nuclei (scores 0.9 and 1.1 (Figure 1, Row **A**, Column **Q**), with only the LC and PBN being more affected (Figure 1, Row **A**, Column **J**; Figure 4 **I–P**). In terminally ill NFH‐Cre/M*lox*P mice (ME7, 28 wpi; RML, 35 wpi; (Figure 1, Rows **C**,**F**), where pathology was most severe, the pattern of abnormal PrP deposition was very similar in both groups (Figure 4 **Q–X**). In comparison with non‐recombined mice (Figure 1, Rows **C**,**F**), NFH‐Cre/M*lox*P mice (Figure 1, Rows **B**,**E**) showed less intense deposition of abnormal prion protein, except in the LC, the NTS and the cerebellum, where more abnormal PrP accumulated (Figure 1, Columns **J**,**M**,**H**). Spongiosis and gliosis were widespread but with different degrees in different brainstem nuclei. We also observed abnormal PrP deposition in the tracts of the cranial nerves (Figure S2C,D) and in the Virchow–Robin spaces and in the brain parenchyma directly surrounding them (Figure S2E,F). This feature was absent in terminally ill M*lox*P mice, raising the possibility that it was an effect of prolonged production of abnormal prion protein due to increased survival in (recombined) NFH‐Cre/M*lox*P mice.

### Semi‐quantitative cross‐regional assessment of spongiosis, gliosis and PrP deposits reveals differential patterns of prion pathology progression in brainstem nuclei

The LC, the NTS and the PBC, identified as first areas of prion protein accumulation, have an important role in the control of respiratory and cardiac activity. We hypothesized that their cellular impairment may correlate with clinical onset and they could indeed be relevant candidates for clinical target areas of prion disease. Following this initial phase, the kinetics of pathology progression in these nuclei in ME7‐inoculated M*lox*P and NFH‐Cre/M*lox*P shows a sharp rise in PrP deposition in the LC, olive and the NTS initially, followed by other areas ‘catching up’ with prion protein deposition towards the late disease stage. In RML‐inoculated animals, the LC is most affected, followed by TN and PBN. To generate a comprehensive map (Figure 1) of the pathologies ‘spongiosis, PrP deposition and gliosis’, we computed the average scores of all nuclei in at least three experimental animals. Visualization using a ‘heatmap’ algorithm reveals early onsets and regional differences that are in addition quantified by forming average values and their standard deviations. We show here that in early stages spongiosis, gliosis and PrP develop more synchronously in ME7 (SD of scores 0.39) than in RML‐inoculated animals (SD of scores 0.61) (Figure 1 **A**,**D**). Later during disease progression, the overall SD equalizes between the two inoculum groups, confirming the visual impression that the spongiosis, gliosis and PrP deposition ‘saturate’.

### The LC (Figure 1, Column **J**; Figure 5)

**Figure 5:**
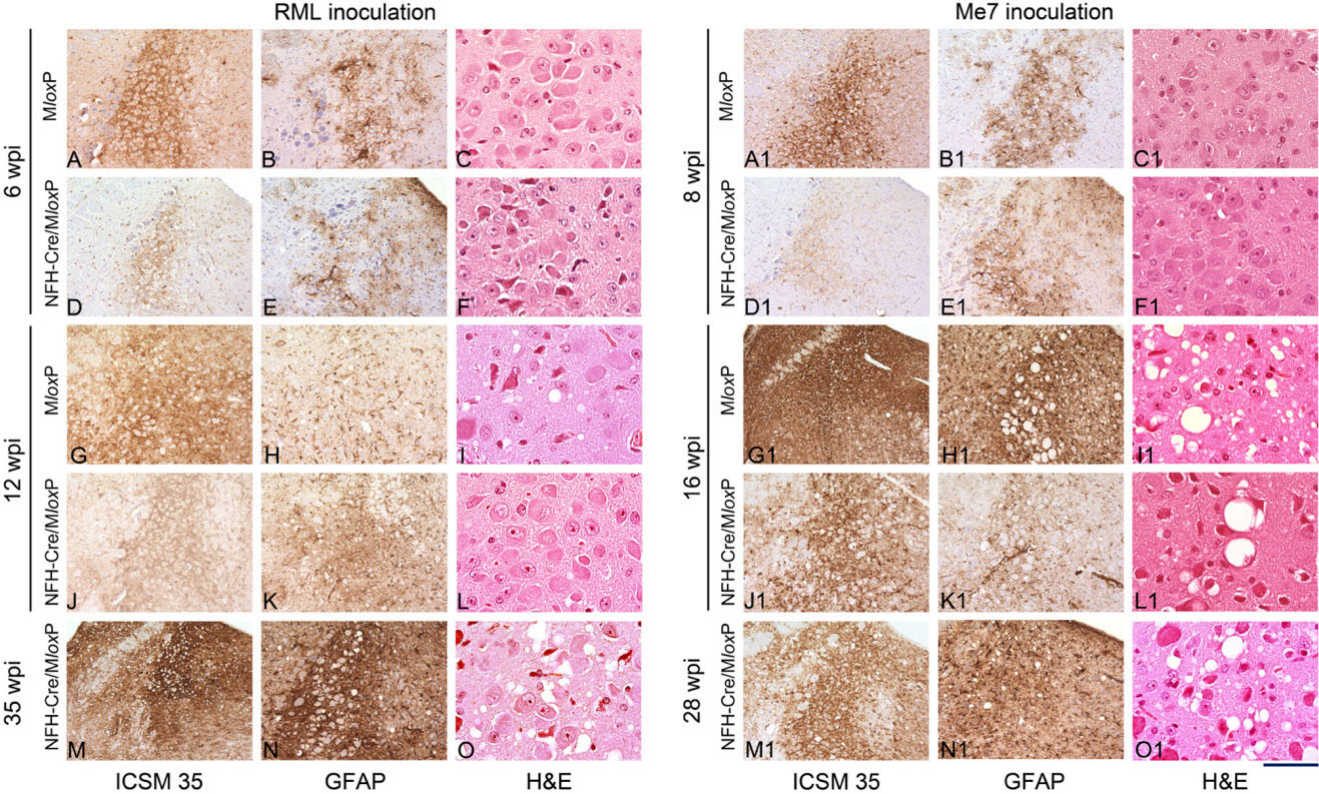
Progression of prion pathology in the locus coeruleus of RML‐ and ME7‐inoculated M*lox*P and NFH‐Cre/M*lox*P mice. Sections were immunostained with ICSM35 antibody for abnormal PrP deposition (left column; RML: **A**,**D**,**G**,**J**,**M**; ME7: **A1**,**D1**,**G1**,**J1**,**M1**), anti‐GFAP antibody for detection of astrocytosis (middle column; RML: **B**,**E**,**H**,**K**,**N**; ME7: **B1**,**E1**,**H1**,**K1**,**N1**), and stained with haematoxylin and eosin (H&E) to assess spongiform changes (right column; RML: **C**,**F**,**I**,**L,O**; ME7: **C1**,**F1**,**I1**,**L1**,**O1**). **A–O** Pathology in RML inoculated brains: at 6 wpi, there is synaptic PrP^Sc^ deposition in M*lox*P mice (**A**), or a mild granular deposition in depleted NFH‐Cre/M*lox*P mice (**D**). No difference in gliosis (**B**,**E**) and no spongiosis (**C**,**F**). At 12 wpi (**G–L**), strong accumulation in the LC of terminally ill M*lox*P mice (**G**), but not in NFH‐Cre/M*lox*P mice was seen. Gliosis was stronger in NFH‐Cre/M*lox*P (**K**) mice than in control M*lox*P mice (**H**). Spongiform changes in M*lox*P mice (**I**) but not in NFH‐Cre/M*lox*P mice (**L**). **M–O** terminally ill NFH‐Cre/M*lox*P mice showed accumulation of PrP, gliosis and spongiosis. **A1–O1** Pathology in ME7‐inoculated brains: At 8 wpi the abnormal accumulation in the LC of M*lox*P mice was intermediate (**A1**) and less intense in NFH‐Cre‐M*lox*P mice (**D1**) and there was mild gliosis and no spongiosis (**B1**,**C1**,**E1**,**F1**). At 16 wpi, M*lox*P mice were terminally ill and showed strong PrP accumulation, gliosis and spongiosis in and beyond the LC (**G1**,**H1**,**I1**), whereas asymptomatic NFH‐Cre‐M*lox*P were less affected (**J1**,**K1**,**L1**). In terminally ill NFH‐Cre‐M*lox*P mice, there was severe LC pathology (**M1**,**N1**,**O1**). Scale bar = 160 μm in all immunostained sections and 80 μm in the H&E stained sections.

At 6 wpi, the deposition in the LC of RML‐inoculated M*lox*P mice was of intermediate intensity (Figure 5 **A**), whereas in the PrP‐depleted NFH‐Cre/M*lox*P mice, it was less pronounced (Figure 5 **D**). At 12 wpi, the LC of terminally ill M*lox*P mice showed strong PrP accumulation (Figure 1 **F**, Column **J**; Figure 5 **D**) and mild spongiosis (Figure 5 **I**) while at the same time the asymptomatic recombined NFH‐Cre/M*lox*P mice showed intermediate levels of PrP accumulation (Figure 1 **D**, Column **J**; Figure 5 **J**) and little spongiosis (Figure 5 **L**), due to the depletion of host PrP^C^. Terminally ill NFH‐Cre/M*lox*P showed severe spongiosis, gliosis and prion protein accumulation (Figure 1 **F**; Figure 5 **M–O**).

ME7‐infected M*lox*P mice showed moderate PrP accumulation at 8 wpi (Figure 5 **A1**), more than in NFH‐Cre/M*lox*P mice (Figure 5 **D1**), whereas gliosis was similar (Figure 5 **B1**,**E1**), and no spongiosis was present (Figure 5 **C1**,**F1**). Terminally ill ME7‐infected M*lox*P mice (16 wpi) showed much heavier PrP accumulation, gliosis and spongiosis than NFH‐Cre‐M*lox*P mice at the corresponding time point (Figure 5 **G1–L1**). Terminally ill NFH‐Cre‐M*lox*P mice (28 wpi) showed strong abnormal PrP accumulation and spongiosis in the LC, whereas the surrounding areas were almost spared from prion accumulation; gliosis was severe in the LC and surrounding nuclei (Figure 1 **C**, Column **J**; Figure 5 **M1–O1**).

### The nucleus of the solitary tract (Figure 1, Column **M**; Figure 6)

**Figure 6:**
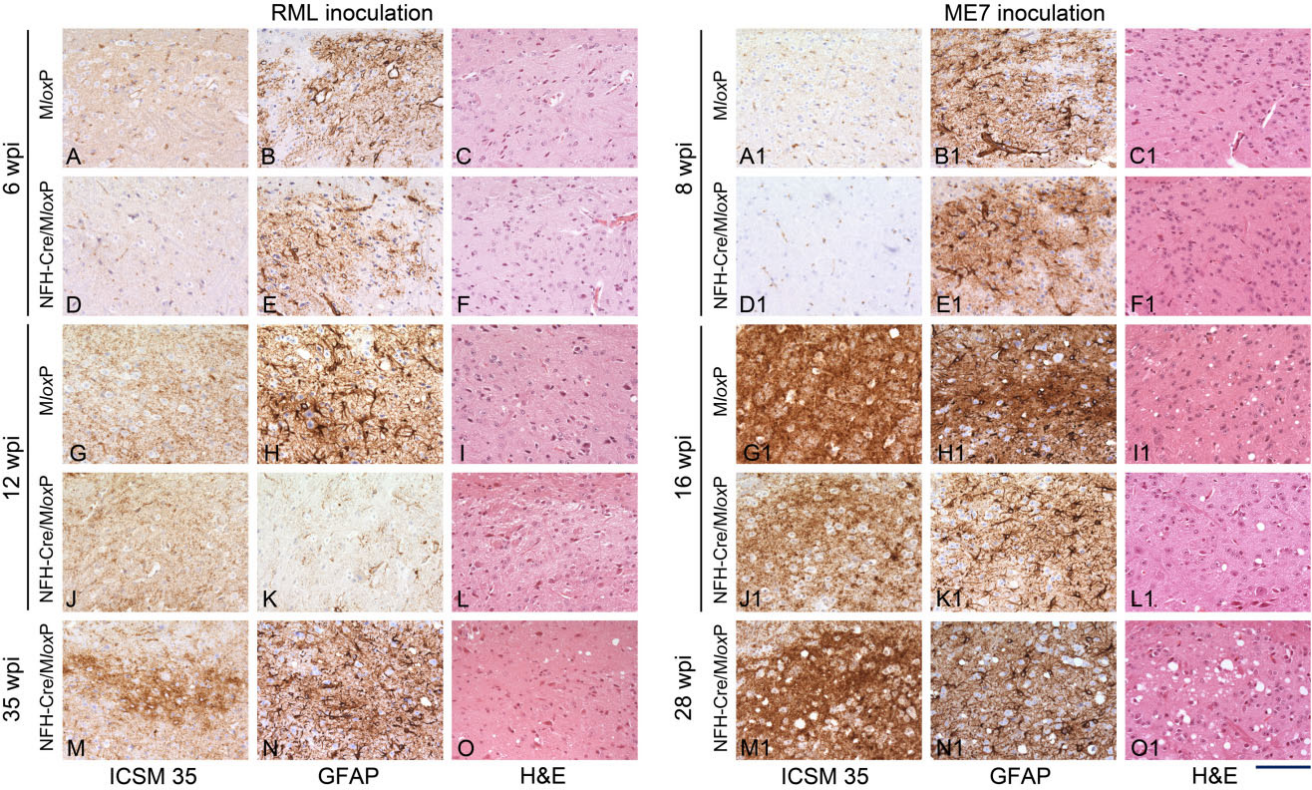
Progression of prion pathology in the nucleus of the solitary tract of RML‐ and ME7‐inoculated M*lox*P and NFH‐Cre/M*lox*P mice. Sections were immunostained with ICSM35 antibody for abnormal PrP deposition (left columns) anti‐GFAP antibody for detection of astrocytosis (middle columns) and stained with haematoxylin and eosin (H&E) to assess spongiform changes (right columns). (**A–O**) RML inoculated M*lox*P mice (6 wpi) showed mild abnormal PrP accumulation, gliosis and no spongiosis (**A–C**). At the same time, NFH‐Cre‐M*lox*P mice showed a similarly mild pathology (**D–F**). At 12 wpi, terminally ill M*lox*P mice (**G–I**) showed more PrP accumulation, strong gliosis and minimal spongiosis. At the same time NFH‐Cre/M*lox*P mice showed similar PrP accumulation but less gliosis and no spongiosis (**J–L**). Terminally ill NFH‐Cre‐M*lox*P mice showed stronger PrP accumulation, gliosis and spongiosis, similar to terminally ill M*lox*P mice (**G–I**). **A1–O1** ME7‐inoculated mice showed minimal PrP deposition, no spongiosis and moderate gliosis at 8 wpi (**A1–F1**) and a rapid progression to a more severe pathology at 16 wpi, when M*lox*P mice are terminal (**G1–L1**). At end stage (**M1–O1**), NFH‐Cre/M*lox*P mice showed severe pathology with abundant PrP, intense gliosis and moderate spongiosis. Scale bar = 160 μm.

At 6 wpi, the NTS of RML‐inoculated M*lox*P mice was affected by mild abnormal PrP accumulation and gliosis (Figure 6 **A**,**B**), whereas in NFH‐Cre/M*lox*P mice there was less abnormal PrP accumulation but a similar degree of gliosis (Figure 6 **D**,**E**). At 12 wpi, PrP deposition, spongiosis and gliosis had increased in M*lox*P mice but not in depleted NFH‐Cre/M*lox*P mice (Figure 6 **G–L**). In terminally ill NFH‐Cre‐M*lox*P mice (35 wpi), reactive gliosis (Figure 6 **N**) was similar to that in terminally ill M*lox*P mice (Figure 6 **H**), and abnormal PrP accumulation was even stronger than in M*lox*P mice (compare Figure 6 **M**,**G**).

ME7‐inoculated mice showed minimal granular deposition of abnormal PrP in the NTS at 8 wpi, a similar degree of gliosis and no spongiosis (Figure 6 **A1–F1**). By 16 wpi, M*lox*P mice showed a substantial aggravation of pathology, with severe PrP accumulation, gliosis and spongiosis, (Figure 6 **G1–I1**), whereas NFH‐Cre/M*lox*P mice showed reduced pathology (Figure 6 **J1–L1**). At the end stage (28 wpi), pathology in NFH‐Cre/M*lox*P mice (Figure 6 **M1–O1**) was similar to 16 wpi M*lox*P mice.

### The PBC (Figure 1, Column **O**; Figure 7)

**Figure 7:**
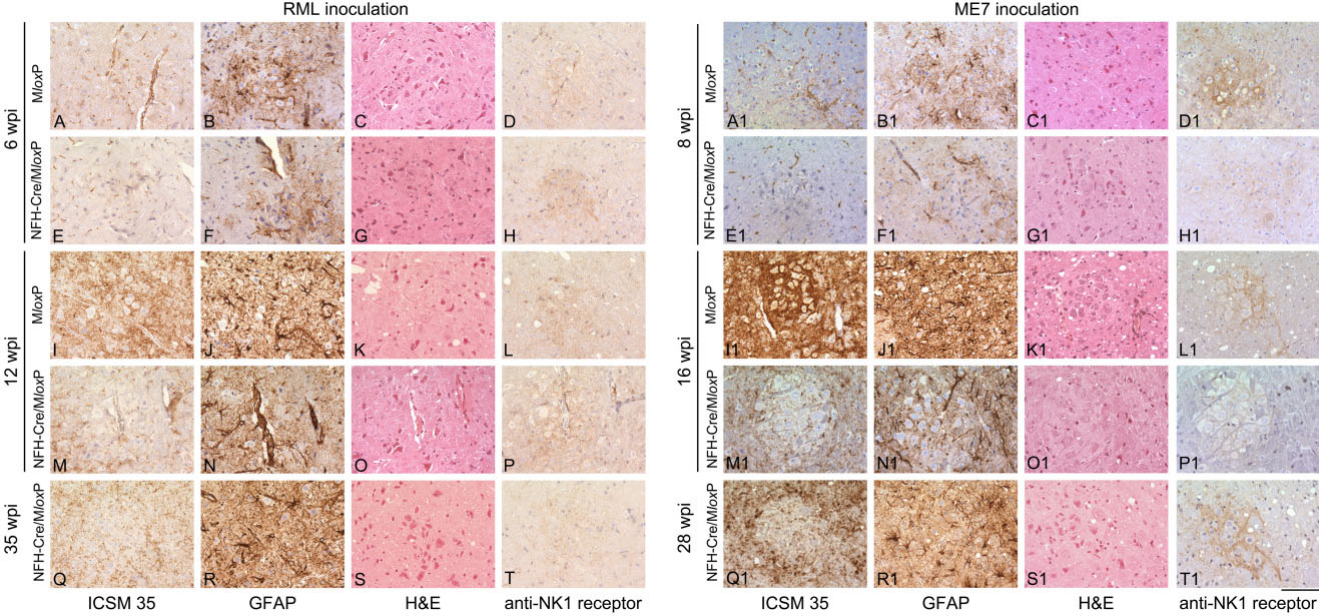
Progression of prion pathology in the pre‐Bötzinger complex of RML‐and ME7‐inoculated M*lox*P and NFH‐Cre/M*lox*P mice. Sections were immunostained with ICSM35 antibody for abnormal PrP deposition (left column) anti‐GFAP antibody for detection of astrocytosis (second column) and stained with haematoxylin and eosin (H&E) to assess spongiform changes (third column) and NK1 Receptor (right column). **A–T** RML‐inoculated M*lox*P mice (6 wpi) showed mild to moderate abnormal PrP accumulation, gliosis and no spongiosis (**A–C**). At the same time, NFH‐Cre/M*lox*P mice showed a similarly mild pathology (**E–G**). At 12 wpi, terminally ill M*lox*P mice (**I–K**) showed more PrP accumulation, strong gliosis and minimal spongiosis. At the same time NFH‐Cre/M*lox*P mice showed similar PrP accumulation but less gliosis and no spongiosis (**M–O**). Terminally ill NFH‐Cre‐M*lox*P mice showed still a moderate PrP accumulation, gliosis and spongiosis, similar to terminally ill M*lox*P mice (**I–K**). There is some inconsistency of progression, as the gliosis in (**E**) is stronger than that in (**K**), indicating a possible variance in transgene recombination efficacy. The right column demonstrates the NK1R immunolabelling of the pre‐Bötzinger complex. **A1–T1** ME7‐inoculated M*lox*P and NFH‐Cre/M*lox*P mice show minimal PrP deposition, no spongiosis and moderate gliosis at 8 wpi (**A1–C1**, **E1–G1**). At 16 wpi more severe pathology, with abundant PrP deposition, severe gliosis and spongiosis was observed in terminally sick M*lox*P mice (**I1**–**K1**) but not asymptomatic NFH‐Cre/M*lox*P mice (**M1**–**O1**). At end stage (**Q1**–**S1**), NFH‐Cre/M*lox*P mice showed severe pathology with abundant PrP, intense gliosis and moderate spongiosis. Right column, NK1R immunolabelling to verify the pre‐Bötzinger complex. Scale bar = 160 μm.

At 6 wpi, the PBC of RML‐infected M*lox*P mice showed mild accumulation and gliosis and no spongiosis (Figure 7 **A–C**). Almost no pathology was seen in NFH‐Cre/M*lox*P mice (Figure 7 **E–G**). Upon disease progression, the effect of recombination became more evident: at 12 wpi, when RML‐infected M*lox*P mice were terminally ill and RML‐infected NFH‐Cre/M*lox*P mice were asymptomatic, M*lox*P mice had more prominent pathology than recombined NFH‐Cre/M*lox*P mice (compare Figure 7 **I–K** and **M–O**). Terminally ill NFH‐Cre/M*lox*P mice (35 wpi) showed the same pathology (Figure 7 **Q–S**) as terminal (12 wpi) M*lox*P mice (Figure 1 **F**).

In ME7‐infected M*lox*P mice, there was mild pathology, similar to NFH‐Cre/M*lox*P mice (Figure 7 **A1–C1** and **E1–G1**). At 16 wpi, terminally ill M*lox*P mice showed strong prion protein deposition, gliosis and spongiosis (Figure 7 **I1–K1**; Figure 1 **C**, average PrP score 2.7), whereas NFH‐Cre/M*lox*P mice showed only mild PrP deposition (Figure 7 **M1–O1**; Figure 1 **A**, average PrP score 1.5). Terminally ill NFH‐Cre/M*lox*P mice (Figure 7 **Q1–S1**) showed the effect of PrP depletion, with levels of reactive gliosis and spongiosis comparable with terminally ill M*lox*P mice but reduced PrP accumulation (Figure 1 **A**, average PrP score 2.0).

In summary, in both RML‐ and ME7‐inoculated M*lox*P and NFH‐Cre/M*lox*P mice, the appearance of spongiosis in the LC, NTS and PBC, which were the first areas of pathology, correlate with the appearance of clinical signs. As neurodegeneration in these nuclei correspond to manifestation of clinical phenotype and because of their fundamental role in control of autonomic function, we speculate that these nuclei may represent clinical target areas of prion disease.

### Analysis of recombination in the LC, the nucleus of the solitary tract and the PBC

**Figure 8:**
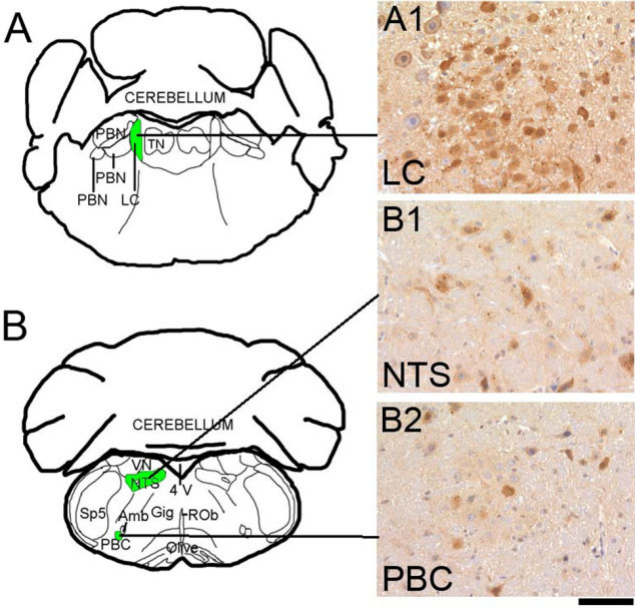
Cre‐mediated recombination in the locus coeruleus (LC), the nucleus of the solitary tract (NTS) and the pre‐Bötzinger complex (PBC) of NFH‐Cre/ROSA26 mice. **A**,**B** schematic representations adapted from the Paxinos Mouse Atlas, coordinates −5.34 mm (**A**) and −6.84 mm (**B**) from Bregma. **A1**,**B1**,**B2** β‐galactosidase immunoreactivity in the LC (**A1**), the NTS (**B1**) and the PBC (**B2**) of NFH‐Cre/ROSA26 mice. Immunostaining for β‐galactosidase is characterized by a diffuse accumulation in the cytoplasm as well as few small intracellular cytoplasmic inclusions. In the LC, ca. half of the cells express β‐galactosidase, indicating partial recombination (**A1**). In the nucleus of the solitary tract (**B1**) and in the pre‐Bötzinger complex (**B2**), recombination was less widespread. Scale bar = 80 μm.

PrP‐depleted NFH‐Cre/M*lox*P mice survive significantly longer than non‐recombined M*lox*P mice but accumulate prion protein and infectivity (Figures 1,2,4, 5, 6). This is reflected in the heavy accumulation of prion protein in the brainstem of NFH‐Cre/M*lox*P mice. At their asymptomatic stages, LC, NTS and PBC were the first areas to accumulate prion protein and to develop spongiform changes. Sustained survival of NFH‐Cre/M*lox*P mice is due to PrP deletion in specific neuronal populations [26]. We therefore analysed the Cre‐mediated recombination in the brainstem, by crossing NFH‐Cre mice with ROSA26*^lox^* reporter mice, which express β‐galactosidase upon Cre‐mediated removal of a stop codon between the ROSA26 promoter and the lacZ gene [30]. Brainstem sections from NFH‐Cre/ROSA26 mice encompassing the LC, the NTS and the PBC were stained with an anti‐β‐galactosidase antibody. In this reporter strain, β‐galactosidase diffusely accumulates in the cytoplasm and typically also forms small intracellular cytoplasmic inclusions that can be visualized by immunostaining. In the LC, we found that approximately half of the cells accumulate β‐galactosidase, indicating partial recombination. In the NTS and PBC, recombination was less frequent but still evident (Figure 8 **A1–B2**). These data suggest that Cre‐mediated recombination in the target areas was incomplete, which could explain why the LC, NTS and PBC ultimately developed prion pathology.

## Discussion

We studied the disease progression in the brainstem of prion‐infected mice in PrP overexpressing M*lox*P and PrP‐depleted NFH‐Cre/M*lox*P mice to evaluate the clinical target areas of prion disease. Signs of functional impairment of the brainstem in humans are vertigo, nystagmus (inferior vestibular nucleus and inferior cerebellar peduncle), nausea and vomiting (area postrema), dysarthria and dysphonia (nucleus ambiguus), singultus (respiratory centre of the reticular formation), ipsilateral flaccid hypoglossal nerve palsy, hemiplegia (contralateral to lesion) with Babinski's sign (contralateral to lesion) and posterior column hypaesthesia (that is hypaesthesia to touch and pressure, with impaired position sense). Syndrome of the caudal base of the pons present with ipsilateral abducens palsy and nuclear facial palsy, analgesia, thermanaesthesia, and impairment of touch, position and vibration sense. Although only selected clinical signs can be monitored in mice with progressive prion disease, a number of key brainstem signs could be mapped to the nuclei that showed early and most progressive prion pathology, including early (clumsiness or lack of coordination, insomnia) and late symptoms in sporadic and iatrogenic CJD (visual deterioration and eventual blindness, myoclonus and paralysis, slurred speech, difficulty swallowing and incontinence). Here, prion pathology first occurs in the thalamus and brainstem in PrP overexpressing RML‐inoculated [27] and ME7‐inoculated mice (Figure S3) and subsequently spreads to other brain areas. The first brainstem target areas of prion pathology are the LC, NTS and PBC. Due to Cre‐mediated recombination and removal of neuronal PrP^C^, prion‐infected NFH‐Cre/M*lox*P mice survive two‐ to threefold longer than M*lox*P mice, depending on the prion strain (RML or ME7). Although still significantly prolonged, the survival in RML‐inoculated NFH‐Cre/M*lox*P mice was shorter than seen previously, thought to be caused by a shift of recombination efficiency and timing in this line over multiple generations [26]. However, ultimately, terminal ill mice all show severe spongiosis in the LC, NTS and PBC. The rate of progression of pathology in these nuclei is slower in NFH‐Cre/M*lox*P mice because of partial neuronal PrP^C^ removal through Cre‐mediated PrP excision. As determination of PrP^C^ via biochemical or immunohistochemical means would be inaccurate due to the variability between adjacent anatomical areas or even between individual cells in an area, we used a high‐resolution histological assessment of recombination using NFH‐Cre mice crossed with the ROSA26*^Lox^* ^P^ reporter mouse strain [30] to assess efficacy of recombination in the postulated target areas. Indeed, by determining the recombination using the surrogate marker β‐galactosidase in NFH‐Cre/ROSA26*^Lox^* ^P^ mice, we show that Cre‐mediated recombination is incomplete in these areas (Figure 8), and hence NFH‐Cre/M*lox*P mice are not completely spared by prion pathology.

Key clinical target areas of prion disease have been defined as critical vulnerable areas, in which prion‐induced neurodegeneration must occur for clinical disease to develop [23]. A strain associated tropism have been suggested and may explain the difference in PrP colonization dynamics and anatomical involvement in RML‐ and ME7‐inoculated mice [33, 34]. The vital role of the LC, NTS and PBC in controlling autonomic functions suggests that neurodegeneration of these areas are likely to contribute to the development of clinical symptoms. The LC is implicated in autonomic activities, such as control of respiration [35, 36, 37] and cardiovascular function [38], in keeping with symptoms of terminally ill CJD patients. Specifically, LC neurones participate in the central respiratory network [39] and exhibit chemosensitive signalling behaviour [40, 41]. Pathological involvement of the LC is also known in Rett syndrome [42], and multiple system atrophy (MSA) [43].

The nucleus of the solitary tract is a relay nucleus for integration and modulation of autonomic activity. Many NTS neurones have respiratory‐related activity and central respiratory modulation [44, 45, 46], detect changes in pCO2 in vivo, respond directly to acid in vitro [47] and respond to arterial baroreceptors [48, 49, 50]. It has been suggested that NTS neurones may have a role in impaired ventilator response MSA when activated by hypoxia [51]. The NTS is one of the first areas of prion accumulation in a model of oral infection, thought to ascend via the dorsal motor nucleus of the vagal nerve [52]. Our findings suggest that the NTS is also a particularly vulnerable area upon intracranial inoculation. Autonomic disturbances are key clinical signs in terminal CJD.

*PBC* neurones are characterized by neurokinin 1 receptor immunoreactivity [53]. We took advantage of this property to confirm the identity of the group of cells showing early abnormal PrP accumulation in the ventral medulla. The PBC is the centre of respiratory rhythm generation [54, 55, 56, 57, 58], and ex vivo studies of abolishing PBC function stops rhythm generation [58]. Impairment of PBC function plays a role in Rett syndrome [59], sudden infant death syndrome [60] and MSA [51]. The role of these nuclei (LC, NTS and PBC) in controlling respiratory and cardiac activity and their early involvement in prion pathology suggest they could represent clinical target areas of prion disease. In both RML‐and ME7‐inoculated NFH‐Cre/M*lox*P mice (that is mice with reduced PrP expression), LC and NTS showed severe PrP accumulation, spongiosis and gliosis. In our model, Cre‐mediated recombination in the clinical target areas (LC, NTS and PBC) is incomplete, and this could explain that these nuclei ultimately succumb to prion pathology.

Although previous work investigated the kinetics of prion accumulation in the brain of hamsters and transgenic mice expressing hamster PrP [61], to our knowledge, this is the first detailed histopathological analysis of prion disease in the brainstem of experimental mice. However, prion disease‐related pathology in nuclei involved in control of autonomic functions has been reported for different experimental and naturally occurring prion diseases. In hamsters, orally infected with 263K prions, the dorsal motor nucleus of the vagus nerve followed by the NTS were found to be the first target areas of prion pathology [52]. Involvement of the vagus nerve and the NTS has been reported also in natural and experimental cases of BSE in cattle, confirming the vagal parasympathetic fibre as a route for spreading of prion from periphery to the CNS [62, 63]. Bradycardia, as an effect of increased vagal tone [64, 65] and heart rate variability [66], has been reported in cattle orally challenged with BSE, but ECG variability does not correlate with evidences of heart disease [67]. In a murine model of BSE, early vulnerability of the central serotonergic system was supported by behavioural and anatomical‐pathological observation [68]. Moreover, in BSE‐infected cattle, spongiform lesions and abnormal PrP accumulation was reported in the auditory brainstem nuclei and associated with clinical dysfunction of the auditory system, providing a rationale to use auditory dysfunction as a test for *antemortem* BSE diagnosis [69].

In summary, we followed the progression of prion pathology in the motor and autonomic centres of the brainstem in mice intracerebrally inoculated with prions and found that LC, NTS and PBC are areas of early prion deposition. As their degeneration corresponds to the manifestation of the clinical phenotype and because of their fundamental role in control of autonomic function, we hypothesize that these nuclei play a role in target areas of prion disease, in particular in the view of the significant overlap to neurological signs in human sporadic CJD.

## Author contributions

Ilaria Mirabile: conducted, executed and analysed the experiments, prepared figures and drafted manuscript. Parmjit Jat: molecular biology support and expertise. Sebastian Brandner: histology analysis and expertise and wrote the manuscript. John Collinge: conception and hypothesis, general guidance and funding.

## Supporting information


**Figure S1.** Scoring system used to evaluate prion pathology in the brainstem of prion inoculated animals. Spongiosis was scored considering the ratio between healthy tissue and vacuoles in a given nucleus, observed by H&E staining, as mild = 1 (A), intermediate = 2 (B) or severe = 3 (C). Abnormal PrP accumulation was scored according to synaptic density of abnormal PrP deposits (D, E, F) or granularity (G, H, I), as observed by ICSM 35 antibody staining. Synaptic density was scored as mild (D), intermediate = 2 (E) or severe = 3 (F); granularity was scored as mild = 1 (G), intermediate = 2 (H) or severe = 3 (I). Gliosis was scored taking into account proportion of reactive cells and the intensity of GFAP staining, as mild = 1 (J), intermediate = 2 (K) or severe = 3 (L). Scale bar = 160 μm.
**Figure S2.** Abnormal PrP accumulation in the cranial nerves and in the Virchow‐Robin space of RML and ME7 inoculated NFH‐Cre/M*lox*P mice. In RML and ME7 inoculated NFH‐Cre/M*lox*P mice PrP deposition was also found in areas spared in RML inoculated M*lox*P mice, like the tracts of the cranial nerves (A, B, C, D) and the Virchow‐Robin spaces (A, B, E, F), suggesting that the prolonged survival allows the spread of prions in areas not primarily targeted by the infection, and highlighted the similarity in the lesion profiles of RML and ME7 inoculated NFH‐Cre/M*lox*P mice. Scale bar = 2 mm (A, B); 60 μm (C–F).
**Figure S3.** Progression of abnormal PrP deposition in the brains of ME7 inoculated M*lox*P and NFH‐Cre/M*lox*P mice. (A) It has been previously shown that in RML inoculated M*lox*P mice abnormal PrP deposition accumulates at 6 wpi in the brainstem. By 8 wpi it spreads to the hippocampus and the thalamus, than to the cortex (10 wpi) and by terminal stage it is diffuse in the whole brain (adapted from [30]). (B) ME7 inoculated M*lox*P and NFH‐Cre/M*lox*P mice were time culled at different times post inoculation (wpi). 3 brains per group were analysed.Abnormal PrP deposition in ME7 inoculated M*lox*P mice was first found at 8 wpi, in the thalamus and the brainstem; by 10 wpi it spread to the cortex and hippocampus and became more intense in the thalamus and brainstem; by 16 wpi it involved the cerebellum. At the end stage of the disease, (approximately 17 wpi) abnormal PrP accumulation was widespread in all brain areas, with maximal intensity in the thalamus and brainstem. In NFH‐Cre/M*lox*P mice, the pattern of PrP deposition at 8 wpi was similar to that observed in M*lox*P mice, but at 10 wpi the abnormal PrP deposition was milder. At 12 wpi, intense staining was localized in the thalamus and brainstem; at approximately 16 wpi deposition spread to cerebellum, striatum, and cortex, but the thalamic nuclei, which were the first area of accumulation, and the hippocampus were spared. At 20 wpi the areas of maximal accumulation were: cortex, caudo‐putamen, frontal thalamus, posterior hippocampus, brainstem, and cerebellum. At the end stage of disease frontal hippocampus and ventral thalamic nuclei showed milder abnormal accumulation compared to end stage M*lox*P mice. Brains were stained with antibody ICSM35, scale bar = 1.7 mm for brains labelled with *; 2.2 mm for all the other brains.
