# Supplementary figures and images for: Identification of clinical target areas in the brainstem of prion‐infected mice
Source: Neuropathol Appl Neurobiol. 2015 Apr 23;41(5):613–30. doi: 10.1111/nan.12189 (PMC4949711; doi:10.1111/nan.12189)

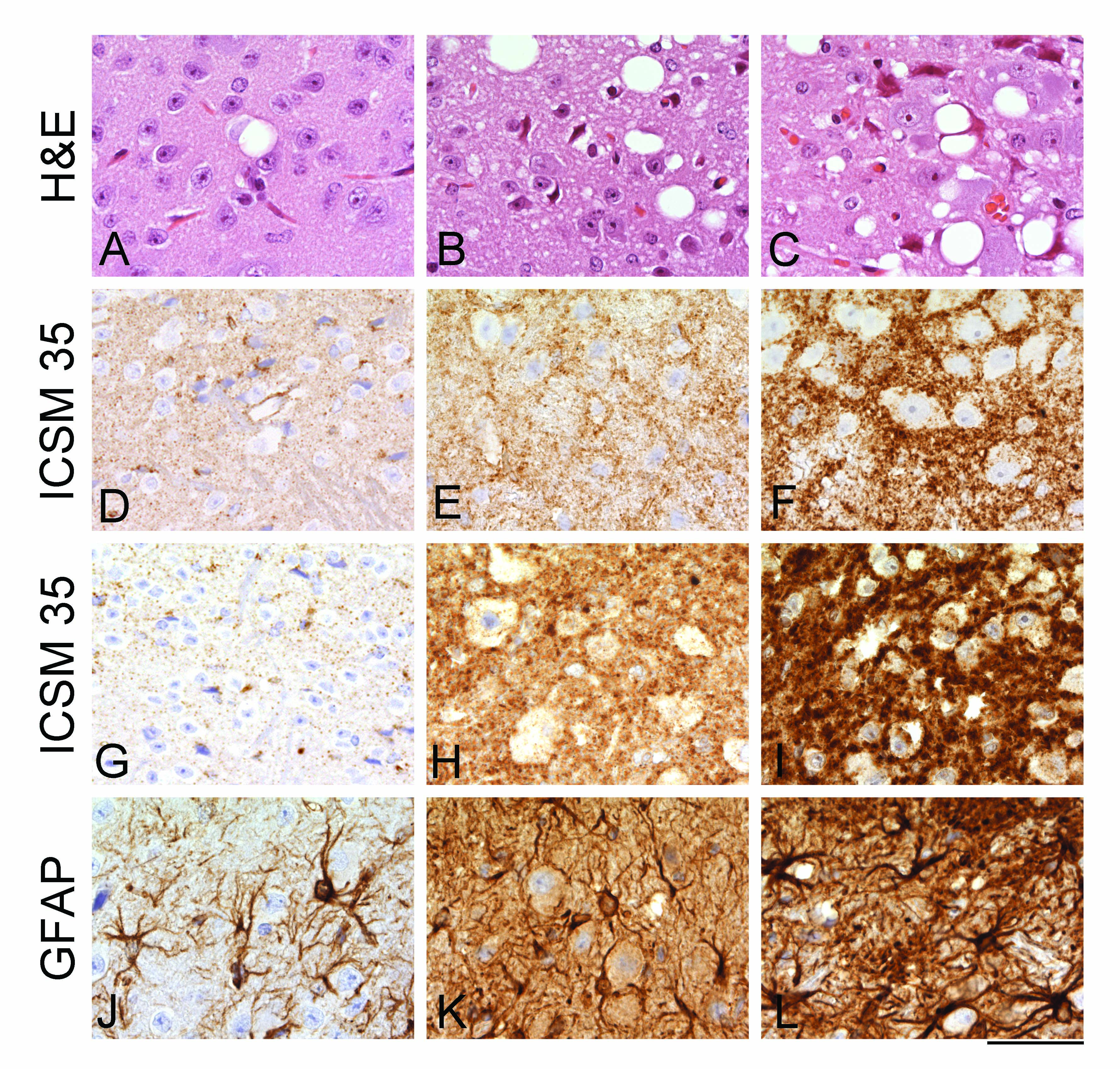

Supplement: Supplementary file 1 — Figure S1. Scoring system used to evaluate prion pathology in the brainstem of prion inoculated animals. Spongiosis was scored considering the ratio between healthy tissue and vacuoles in a given nucleus, observed by H&E staining, as mild = 1 (A), intermediate = 2 (B) or severe = 3 (C). Abnormal PrP accumulation was scored according to synaptic density of abnormal PrP deposits (D, E, F) or granularity (G, H, I), as observed by ICSM 35 antibody staining. Synaptic density was scored as mild (D), intermediate = 2 (E) or severe = 3 (F); granularity was scored as mild = 1 (G), intermediate = 2 (H) or severe = 3 (I). Gliosis was scored taking into account proportion of reactive cells and the intensity of GFAP staining, as mild = 1 (J), intermediate = 2 (K) or severe = 3 (L). Scale bar = 160 μm. Figure S2. Abnormal PrP accumulation in the cranial nerves and in the Virchow‐Robin space of RML and ME7 inoculated NFH‐Cre/MloxP mice. In RML and ME7 inoculated NFH‐Cre/MloxP mice PrP deposition was also found in areas spared in RML inoculated MloxP mice, like the tracts of the cranial nerves (A, B, C, D) and the Virchow‐Robin spaces (A, B, E, F), suggesting that the prolonged survival allows the spread of prions in areas not primarily targeted by the infection, and highlighted the similarity in the lesion profiles of RML and ME7 inoculated NFH‐Cre/MloxP mice. Scale bar = 2 mm (A, B); 60 μm (C–F). Figure S3. Progression of abnormal PrP deposition in the brains of ME7 inoculated MloxP and NFH‐Cre/MloxP mice. (A) It has been previously shown that in RML inoculated MloxP mice abnormal PrP deposition accumulates at 6 wpi in the brainstem. By 8 wpi it spreads to the hippocampus and the thalamus, than to the cortex (10 wpi) and by terminal stage it is diffuse in the whole brain (adapted from [30]). (B) ME7 inoculated MloxP and NFH‐Cre/MloxP mice were time culled at different times post inoculation (wpi). 3 brains per group were analysed.Abnormal PrP deposition in ME7 inoculated MloxP mi [file NAN-41-613-s001.zip › NAN_12189-supp-0001-S1.jpg]

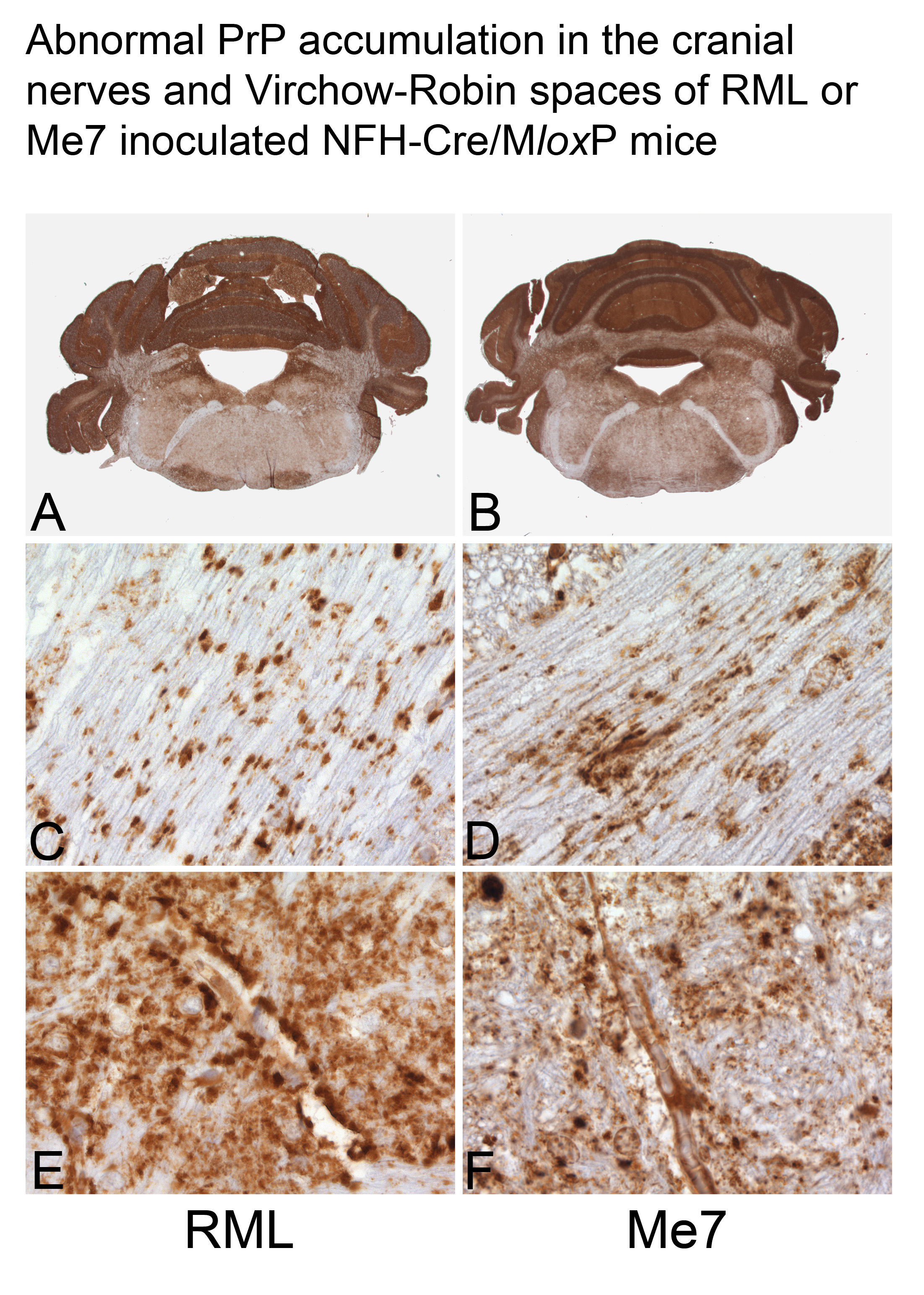

Supplement: Supplementary file 1 — Figure S1. Scoring system used to evaluate prion pathology in the brainstem of prion inoculated animals. Spongiosis was scored considering the ratio between healthy tissue and vacuoles in a given nucleus, observed by H&E staining, as mild = 1 (A), intermediate = 2 (B) or severe = 3 (C). Abnormal PrP accumulation was scored according to synaptic density of abnormal PrP deposits (D, E, F) or granularity (G, H, I), as observed by ICSM 35 antibody staining. Synaptic density was scored as mild (D), intermediate = 2 (E) or severe = 3 (F); granularity was scored as mild = 1 (G), intermediate = 2 (H) or severe = 3 (I). Gliosis was scored taking into account proportion of reactive cells and the intensity of GFAP staining, as mild = 1 (J), intermediate = 2 (K) or severe = 3 (L). Scale bar = 160 μm. Figure S2. Abnormal PrP accumulation in the cranial nerves and in the Virchow‐Robin space of RML and ME7 inoculated NFH‐Cre/MloxP mice. In RML and ME7 inoculated NFH‐Cre/MloxP mice PrP deposition was also found in areas spared in RML inoculated MloxP mice, like the tracts of the cranial nerves (A, B, C, D) and the Virchow‐Robin spaces (A, B, E, F), suggesting that the prolonged survival allows the spread of prions in areas not primarily targeted by the infection, and highlighted the similarity in the lesion profiles of RML and ME7 inoculated NFH‐Cre/MloxP mice. Scale bar = 2 mm (A, B); 60 μm (C–F). Figure S3. Progression of abnormal PrP deposition in the brains of ME7 inoculated MloxP and NFH‐Cre/MloxP mice. (A) It has been previously shown that in RML inoculated MloxP mice abnormal PrP deposition accumulates at 6 wpi in the brainstem. By 8 wpi it spreads to the hippocampus and the thalamus, than to the cortex (10 wpi) and by terminal stage it is diffuse in the whole brain (adapted from [30]). (B) ME7 inoculated MloxP and NFH‐Cre/MloxP mice were time culled at different times post inoculation (wpi). 3 brains per group were analysed.Abnormal PrP deposition in ME7 inoculated MloxP mi [file NAN-41-613-s001.zip › NAN_12189-supp-0002-S2.jpg]

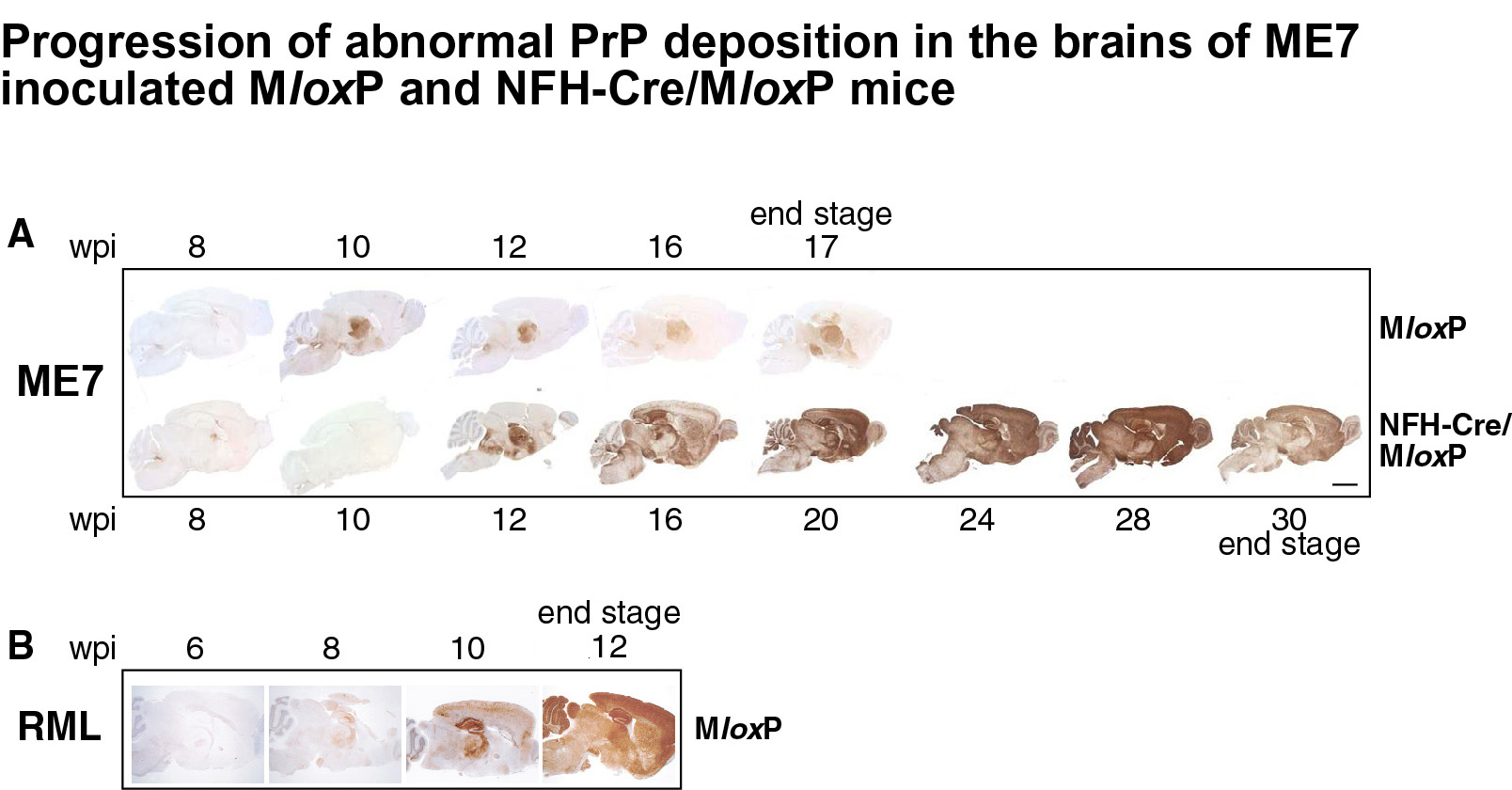

Supplement: Supplementary file 1 — Figure S1. Scoring system used to evaluate prion pathology in the brainstem of prion inoculated animals. Spongiosis was scored considering the ratio between healthy tissue and vacuoles in a given nucleus, observed by H&E staining, as mild = 1 (A), intermediate = 2 (B) or severe = 3 (C). Abnormal PrP accumulation was scored according to synaptic density of abnormal PrP deposits (D, E, F) or granularity (G, H, I), as observed by ICSM 35 antibody staining. Synaptic density was scored as mild (D), intermediate = 2 (E) or severe = 3 (F); granularity was scored as mild = 1 (G), intermediate = 2 (H) or severe = 3 (I). Gliosis was scored taking into account proportion of reactive cells and the intensity of GFAP staining, as mild = 1 (J), intermediate = 2 (K) or severe = 3 (L). Scale bar = 160 μm. Figure S2. Abnormal PrP accumulation in the cranial nerves and in the Virchow‐Robin space of RML and ME7 inoculated NFH‐Cre/MloxP mice. In RML and ME7 inoculated NFH‐Cre/MloxP mice PrP deposition was also found in areas spared in RML inoculated MloxP mice, like the tracts of the cranial nerves (A, B, C, D) and the Virchow‐Robin spaces (A, B, E, F), suggesting that the prolonged survival allows the spread of prions in areas not primarily targeted by the infection, and highlighted the similarity in the lesion profiles of RML and ME7 inoculated NFH‐Cre/MloxP mice. Scale bar = 2 mm (A, B); 60 μm (C–F). Figure S3. Progression of abnormal PrP deposition in the brains of ME7 inoculated MloxP and NFH‐Cre/MloxP mice. (A) It has been previously shown that in RML inoculated MloxP mice abnormal PrP deposition accumulates at 6 wpi in the brainstem. By 8 wpi it spreads to the hippocampus and the thalamus, than to the cortex (10 wpi) and by terminal stage it is diffuse in the whole brain (adapted from [30]). (B) ME7 inoculated MloxP and NFH‐Cre/MloxP mice were time culled at different times post inoculation (wpi). 3 brains per group were analysed.Abnormal PrP deposition in ME7 inoculated MloxP mi [file NAN-41-613-s001.zip › NAN_12189-supp-0003-S3.jpg]
